# The Impact of Trust and the Role of the Opt-Out Mechanism in Willingness to Share Health Data via Electronic Health Records in Germany: Telephone Survey Study

**DOI:** 10.2196/65718

**Published:** 2025-04-15

**Authors:** Felix Wilke

**Affiliations:** 1 Department of Social Work, University of Applied Sciences Jena, Carl-Zeiss-Promenade 2, Jena, 07745, Germany, 49 3641205815

**Keywords:** data sharing, health, citizens, electronic health record, trust, digitalization, opt-out

## Abstract

**Background:**

Electronic health records (EHRs) offer a valuable resource for research and health care improvement. However, public acceptance of sharing personal health data is critical to the success of such initiatives. In Germany, automatic data sharing via EHRs will be implemented unless people opt out.

**Objective:**

This study aims to assess the willingness of the German population to share health data via EHRs and to explore the role of trust in influencing these attitudes.

**Methods:**

A computer-assisted telephone interview study was conducted in December 2023, with 1004 respondents aged 18 years and older, representative of the German population. Descriptive statistics and multivariate linear regression models were used to analyze the data.

**Results:**

The survey shows that 43.4% (n=432) of respondents would be willing to share their health data via EHR, and a significant 34% (n=338) remain undecided. While the population is open to adoption of the EHR for personal health issues (n=483, 53% show interest in using it), the opt-out model for data sharing is viewed critically, with 44.7% (n=438) of respondents rejecting it. Socioeconomic status significantly influences the willingness to share data, with higher income, education, and digital literacy being associated with greater openness to data sharing. However, trust emerged as the most significant factor. Additionally, experiences with digital technologies increase the willingness to share personal health data.

**Conclusions:**

The German population shows general openness toward EHRs and data sharing. Trust plays a critical role in promoting willingness to share health data. The findings highlight challenges in Germany’s transition to an opt-out system.

## Introduction

### Legislative Context and Research Questions

Health data stored in electronic health records (EHRs) are considered an extremely valuable source for advancing medical science and health care. EHRs have the potential to provide high-quality longitudinal data from a broad spectrum of the population. It is therefore not surprising that many countries are implementing data-sharing options to make use of these data. In Germany, the EHR (“elektronische Patientenakte”) was introduced on January 1, 2021, and is available to all legally insured persons through their health insurance company. It can be used to store and manage medical diagnoses and treatment data. The use of the EHR also allows control over who can view and access data. However, by 2024, only 1%‐2% of the eligible population have an EHR. To increase the prevalence and benefits of the EHR, the German Bundestag passed the Digital Act (“Digital-Gesetz”) and the Health Data Use Act (“Gesundheitsdatennutzungsgesetz”) on December 14, 2023. This means that starting in 2025, for all people with statutory health insurance in Germany, an EHR will be automatically created unless they opt out. The Health Data Use Act further allows for the use of pseudonymized health data for research purposes by public and private institutions by amending §303e SGB V. That means data stored in the EHR will be available for research purposes including commercial purposes unless people opt out. The data will be made available through the German Health Research Data Center (“Forschungsdatenzentrum”). The legislative changes mark a paradigm shift in the way health data are handled: the creation of electronic patient records and the sharing of health data will be automatic unless people actively opt out. The German federal minister of health, Karl Lauterbach, stated that the paradigm shift will be a “gamechanger” for the development of health [1]. The new legal framework marks a step toward digitization that many experts have been calling for for a long time [2]. At the same time, the extensive and passive sharing of health data has been criticized by various stakeholders.

This paradigm shift raises some serious questions about the social implications. There are a number of challenges to the “data is gold” perspective [3]. Some social scientists criticize that digitization in health bears potential for “panoptic surveillance” [4]. Other critics [5] have highlighted data security issues that can harm citizens in many ways. Furthermore, the risks, but also the opportunities to benefit from digital health, are unevenly distributed across society. There is ample empirical evidence of a so-called “digital divide,” meaning that people with lower socioeconomic status have lower chances of using digital (health) services and face higher risks [4,6,7]. The digitization of health therefore needs to be critically monitored. Citizens should be aware of the potential benefits as well as the potential dangers of sharing personal health data. This becomes even more urgent with the implementation of an opt-out mechanism. Opt-out is an instrument of nudging [8]. It aims to increase the use of EHRs and the acceptance of data sharing by “choice architecture” [9]. Citizens retain their freedom of choice but are nudged toward using the EHR. However, an opt-out mechanism relies on informed citizens to take deliberative action, especially when data sharing can be harmful. The policy of choice architecture and the use of EHR data for health research make clear that health data sharing cannot be reduced to an individual action but must be analyzed in its social context. It raises questions of legitimacy, social inequality, and the diffusion of knowledge among citizens.

This paper focuses on public opinion on EHR data sharing in Germany. It answers three research questions: (1) Is there sufficient willingness in society to share health data that justifies the introduction of an opt-out scheme? (2) Does the willingness to share data differs with socioeconomic status? (3) How can individual willingness to share EHR data be explained?

### State of the Art

This section summarizes the research on data sharing and evidence on EHR adoption. International research finds widespread support for the idea of EHRs among citizens and institutions in the health care sector [10-12]. However, “evidence indicates that consumers who have been offered access to PEHRs have not widely used them and the number of active users has remained low” [12]. For example, a recent study shows that in the United States, the number of active users of the EHR is about 26% (2019), despite its increasing integration in medical practice [13]. In Germany, until 2024, according to data from public health insurance, less than 2% of the insured actively requested an EHR [14]. At the same time, only about half of the population is even aware of the EHR [15]. Several studies have shown that interest and use of the EHR show a digital divide. Younger and more digital literate people show a higher probability of using EHRs, while older and low-income groups have less often access [12,16,17]. Regarding socioeconomic factors, lower educational and employment status, as well as belonging to an ethnic minority, have a negative impact on individuals’ desire to access and use EHRs [12,18].

Several studies have examined citizens’ motivations for using EHRs. The perceived advantages and disadvantages strongly influence the intention to use and the actual use. Key factors here are concerns about data protection and the fear of being discriminated by health insurers or employers on the one hand, and the benefits of better information and experience with the portals or providers on the other [12,19,20]. Data protection concerns are a key factor because health issues are relevant in many areas of daily life [10,12,19]. This can be seen directly when respondents report fears of stigmatization as a result of digitally disseminated health information from the EHR [21,22]. For people with chronic diseases, the balance of advantages and disadvantages seems to be clearly in favor of the advantages. Several studies have reported higher levels of interest in providing data due to the hope of treatment progress [23,24]. For people with chronic diseases, there are already care programs in place that people can benefit from, such as disease management programs [25].

While the research literature stresses that EHRs are potentially an important tool to transform “passive recipients of treatment to empowered consumers” [12], the empirical findings are rather disappointing. Several international studies have shown that the overall use of EHRs by patients remains low [12]. Furthermore, there is little evidence of empowerment effects [12]. Even people who have an EHR often do not know which data are stored in the health record and how to interpret the information [12,26]. When users experience that they do not understand the EHR or the information stored in it, their willingness to use it decreases significantly [23]. Thus, we can expect a significant gap between interest in EHRs and actual use. This gap is likely to be even more pronounced in Germany, with its high standards for data security and complicated routines for accessing the EHR.

Data sharing is an essential part of EHRs. Many EHR systems allow anonymized health data to be used for scientific research. While this can lead to progress in the health system, there is no clear-cut advantage for an individual sharing its data, since anonymization makes individual feedback regarding new health insights hard. That might be the reason, why in public discourse, data sharing is often labeled as “data donation” [27]. Research regarding data sharing has shown that a considerable part of the population (often exceeding 50%) is willing to share its health data [27-30]. There are considerable differences between different countries. For example, the Scandinavian countries show the highest willingness to share health data, while people in Southern Europe are rather skeptical about it [31]. For Germany, recent research suggests that 8 of 10 citizens are willing to share their health data for scientific purposes [15,32,33]. Notably, this high approval rate also seems to hold true for the planned opt-out regulation [33]. However, these high approval rates have to be interpreted with caution, since limited knowledge about health data sharing is widespread and some studies have methodological limitations. Like many other surveys in this area, the high approval rates rely on web-based surveys that have proven to be of less quality regarding the research results compared to face-to-face or telephone interviews [34]. One reason is that they show a higher tendency to produce biased results [35]. Since there is an empirical correlation between digital literacy and the willingness to share health data, the results are likely to be too optimistic. Furthermore, some questionnaires tend to highlight the positive impacts of data sharing and thereby inflating the approval rate (The question of the survey conducted in Germany was [15,32]: “Assuming that in the future your personal health data, such as your medical history, examination results, X-ray images, etc. can be stored online in a digital health record. Would you agree that your personal health data is used anonymously and free of charge for the purpose of medical research so that in the future diseases can be better recognized and new treatments developed?” And “At present, the use of patient data for medical research requires the consent of patients. However, it is often difficult or even impossible to obtain such consent. Therefore, there are considerations in Germany to legally allow medical research without consent on encrypted patient data after an independent review of the research project. In return, it should be possible for every citizen to simply object to this so-called ‘data donation’ How would you find such a legal regulation ... for publicly funded research?”).

Empirical studies have revealed that the willingness to share data differs among different groups. The findings are comparable to the overall use of EHR: among those with higher age, educational level, and income, a higher willingness to share data can be observed [30,36-39]. Some studies investigate potential individual causes for the willingness to share health data for scientific purposes. These studies find that concerns about confidentiality breaches and data misuse are important factors that prevent data sharing [28,29]. Unsurprisingly, privacy protection, anonymization, and transparency have proven to be key factors for data sharing [29,40]. Furthermore, people are motivated by potential benefits, including more altruistic ones, like advancements in science and public health [29,40]. Another key factor is the use of health data. While international research has shown relatively high public support for the secondary use of health data, this support decreases significantly when shared data can be used by the private sector [41-43].

Another source of insight comes from studies that focus on the social contexts in which data are shared. They have identified 2 other relevant aspects: digital literacy and trust. Health data sharing relies on digital tools. Therefore, people who are more familiar with digital tools may be less reluctant to share data. In addition, technology companies such as Google and Apple have created many services that make use of data sharing in everyday life. Hence, digitally literate people are more involved in data sharing in general. In line with this argument, empirical studies show a higher propensity to share health data among the more digitally savvy people [36,37,40,44,45]. On a more abstract level, it can be argued that trust plays an essential role in health in general [46] and in data sharing in particular. Since the sharing of data is a socially highly indeterminate constellation for social action, trust is a crucial resource. The risks taken by data donors as well as the potential benefits depend strongly on other actors and can hardly be known in advance. For example, the question of anonymizing health data can only be answered in relation to existing knowledge and techniques. The benefits of data sharing are particularly uncertain because there is no guarantee of medical progress, and even if progress does occur, it is unclear whether data donors themselves benefit. Given these theoretical arguments, it is not surprising that empirical studies show an important influence of trust. They can show that trust in the institutions involved in data sharing is relevant, as is general trust in science [15,29,36,37,40,44,45].

## Methods

### Ethical Considerations

Ethics approval was obtained from the ethics department of the German Aerospace Center (21/23) before the interviews were conducted. Participants gave verbal informed consent to participate in the study. Anonymized interview data were provided to the author by the survey institute. No compensation was given to the interviewees.

### Survey Design and Sample

The analysis is based on a dedicated telephone survey (computer-assisted telephone interview [CATI]) about health data conducted between December 7 and 21, 2023. The sampling was carried out by the survey institute drei.fakt (Erfurt) on behalf of the Ernst Abbe University of Applied Sciences Jena. For the study, a representative randomized stratified random sample was drawn based on randomized landline and cell phone numbers (30/70) [47]. Random landline numbers were called based on regional quotas. The mobile phone numbers were sampled and stratified by telephone provider. A total of 11,451 residential numbers were called. In total, 1004 respondents aged 18 years and older participated in an interview. In contrast to many web-based surveys in this area, CATI allows people without internet access to be able to take part in the survey. To ensure the quality of the sample, it was compared with the population for age, gender, and region during the field period. Statistics from the 2019 German microcensus served as a data basis for comparing the distribution of the sample with the distribution of the characteristics in the population. Compared to population statistics, the unweighted sample underrepresents immigrants and slightly overrepresents people with higher education. This selection bias of telephone interviews is well-known from survey research. Therefore, all analyses use a weighting variable in order to adequately represent the population in terms of demographic and socioeconomic characteristics.

### Questionnaire

The questionnaire comprised 32 questions regarding various topics about health data sharing. The questionnaire included information on digital skills, the willingness to share personal health data in general, motives for and against data sharing, questions regarding trust and data protection, as well as attitudes toward the EHR and socioeconomic characteristics. The CATI lasted approximately 20 minutes. Most questions were based on a 5-point Likert scale or binary response options and included multiple response categories. The final questionnaire included feedback from 2 separate pretests. In this pretest, the survey instrument was tested for its practical suitability in the field and potential misunderstandings. The full questionnaire and survey data are available in German via the GESIS repository [47].

### Data Analysis

Descriptive statistics and multivariate linear regression models are used for analysis. The dependent variable in the multivariate regression models is the attitude toward sharing health data via EHR. People were asked on a 5-point scale (1=yes, definitely and 5=no, definitely not): “Would you be willing to share your health data from your EHR anonymized for medical research purposes?” To minimize potential misunderstandings about the EHR, the question was preceded by a brief explanation of EHRs (“Since January 1, 2021, all people with statutory health insurance can use the EHR via an app of the insurance provider. The EHR contains medical findings and information from previous treatments.”). It is important to note that this question is hypothetical, as only a small fraction of people actually had an electronic personal health record at the time of the fieldwork (see Introduction section). However, in order to measure the willingness to share personal health information and to take into account the new legal framework, the question was phrased in a hypothetical manner. This makes it possible to measure the willingness to share extensive and potentially sensitive personal health information rather than actual planned behavior. Therefore, the question is also analyzed for respondents without an EHR in order to reflect overall opinions about the new legislative approach.

## Results

The first step is to analyze the approval rates for the use of the German EHR. In general, the population is very open to the EHR. In total, 53% (n=483) of the population who claim to have no EHR yet show interest in using it. At the same time, 8.9% (n=89) of the sample state that they already have an EHR, in comparison to the 1% reported in official statistics. Therefore, it has to be assumed that some people lack a proper understanding of the EHR (see Limitations and Further Research Questions section). Only a minority of 17.1% (n=156) refuse to use it. The proportion of people with no clear opinion (partly partly or do not know) is significantly higher (n=272, 29.9%).

**Figure 1.**
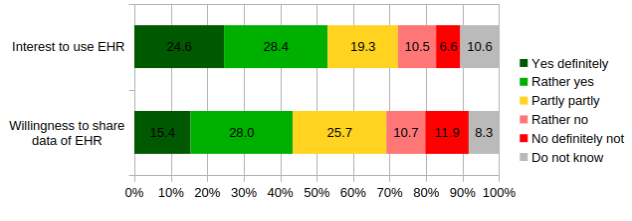
Opinions about the use of electronic health record (EHR) and data sharing for research purposes (in percentages). Own calculation and weighted results for the following questions: “Would you like to use the EHR to view and manage your digital health data?” (n=911; nonresponse and people with EHR excluded) and “Would you be willing to share your health data from your EHR anonymized for medical research purposes?” (n=995; nonresponse excluded).

The legislative proposals also introduce the sharing of health data from the EHR for research purposes, as long as individuals do not actively object. Figure 1 summarizes whether respondents would be willing to share their personal health data stored in an EHR. The figure summarizes the attitudes of respondents with and without EHR. Respondents are more cautious about data sharing compared to the interest in using the EHR. In total, 43.4% (n=432) of respondents are willing to share their health data (yes, definitely or rather yes). Nearly 1 in 4 (n=225, 22.6%) respondents oppose data sharing. In addition, a large proportion of respondents are undecided (partly or not at all or do not know). A high interest in using the EHR corresponds empirically with a high willingness to share health data via EHR (no figure). The bivariate correlation is considerably large with 0.56 (Spearman).

**Figure 2.**
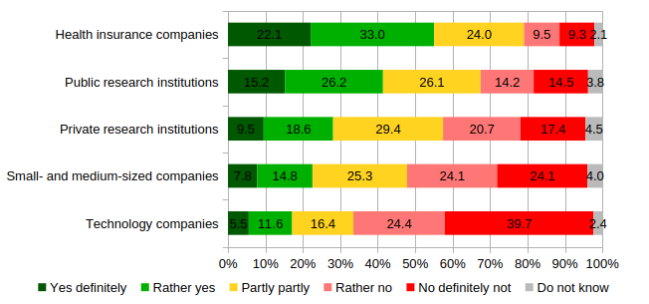
Willingness to share personal health data by data user (in percentages). Own calculation, weighted results for the question, “Would you be willing to share your health data anonymously for medical research purposes with the following institutions?” (nonresponse excluded; n=993).

The attitude toward health data sharing varies considerably with the data user respondents have in mind (Figure 2). The survey included a question regarding health data sharing with different institutions. It has to be noted that this question did not mention the EHR directly but addressed the willingness to share health data more generally. While 28.7% (n=285) and 18.8% (n=186) do not want to share data with public research institutions or public health insurances, about 38% (n=377) object to share their personal health data with private research institutions.

In the next step, the interest to use the EHR and the willingness to share health data for research purposes are measured for different socioeconomic groups. Table 1 shows the percentage of respondents in each group who intend to use the EHR and how many would agree with data sharing (yes and rather yes). In addition, respondents were asked for their opinions on different consent mechanisms. Figure 1 shows the proportion of people who explicitly reject the opt-out procedure as it will be introduced by law in 2025—compared to those who agree or are undecided (do not know).

**Table 1.** Socioeconomic differences in opinions about electronic health record (EHR)^a^.

	Intention to use EHR, n (%)	Willingness to share data of EHR, n (%)	Disagree with opt-out, n (%)
Region			
West Germany	386 (52.1)	350 (43.2)	372 (46.8)
East Germany	97 (57)	82 (44.3)	67 (36)
Gender			
Woman	237 (50.2)	200 (39.1)	228 (45)
Man	245 (56)	232 (47.8)	210 (44.4)
Migrant (not born in Germany)			
No	484 (56.6)	430 (46)	409 (44.5)
Yes	18 (37.3)	18 (33.5)	25 (48.7)
Income quartile			
First (<1250)	67 (48.6)	50 (33.4)	57 (38.5)
Second (<1833)	120 (54.4)	104 (43.9)	90 (38.8)
Third (<2500)	103 (56.5)	97 (49.2)	91 (47.3)
Fourth	134 (61.4)	142 (57.3)	119 (48)
Age (years)			
18‐39	120 (48.8)	116 (41.2)	121 (44)
40‐59	164 (52.7)	140 (42.1)	163 (49.5)
Above 60	201 (56.6)	176 (46.5)	154 (41.2)
Educational level			
Low	99 (51.8)	79 (38.6)	70 (35.3)
Medium	182 (52.9)	172 (46.8)	148 (40.6)
High school degree	84 (49.4)	68 (35.9)	98 (51.9)
University degree	118 (57.7)	116 (50.8)	131 (58.1)
Total sample	483 (53)	432 (43.4)	438 (44.7)
Total respondents, n	911	995	980

a Own calculations, weighted percentages for columns 1 and 2 (share of a sample with statement yes definitely and rather yes) and column 3 (share of a sample who disagrees opt-out) in response to the question: “The German government is planning to use data from the electronic health records for medical research, eg, for research on new diseases and therapeutic approaches. Which of the following consents do you agree with? ... My data will be shared until I object.” Options to answer: agree or disagree or do not know or nonresponse; nonresponse excluded; do not know is interpreted as no interest to use EHR, not in favor of data sharing and agree with opt-out.

There are clear socioeconomic differences in interest in using the EHR and willingness to share health data, which are similar for both variables. The higher a person’s socioeconomic position (like income or education), the more open respondents are to using the EHR and sharing their personal health data for research purposes. Older people and men also tend to be more open. There are pronounced differences by migration status. The final column summarizes attitudes toward the opt-out process. First of all, it is striking that the procedure implemented by the legislator is explicitly rejected by a significant part of the population. In the entire sample, 44.7% (n=438) say that they are against an opt-out. Additional analyses not presented here show that the remaining respondents either agree (n=400, 40.8%) or have no opinion (n=142, 14.5%). Interestingly, the opt-out model is mainly rejected by people in a higher socioeconomic position. There are hardly any differences with regard to the other socioeconomic variables, except for a lower level of rejection in the eastern part of Germany. Overall, the descriptive statistics suggest that citizens are open to EHR and data sharing but want a high degree of autonomy in dealing with their health data. Additional analyses not shown here indicate that active consent is supported by over 80% (n=788) of respondents.

In the next step, the willingness to share data via the EHR is examined in a multivariate linear regression model (Table 2). The dependent variable is the willingness to share personal health data expressed on a 5-point scale (Figure 1). To prevent an excessive reduction in the sample, people who answered “do not know” were assigned to the “partly partly” category. The findings presented here remain stable even with a smaller sample. The determinants identified in the State of the Art section are added as independent variables to the model. Special attention is given to the role of trust. The first model includes socioeconomic parameters. As shown in Table 1, the multivariate model confirms socioeconomic differences. However, some more differentiated statements can be made. Controlling for other parameters, there is a curvilinear relationship between age and willingness to share. The positive squared age term indicates that the willingness to share is highest among the very young and the very old. The willingness to share is lowest among people in their 50s. On a 5-point scale, their willingness is about 0.5 points lower than that of 20-year-old people. Income has a positive significant influence on willingness to share, which confirms the descriptive findings. The level of education also has an influence. However, when controlling for income, medium levels of education are associated with the highest willingness to share. Women and people born abroad show no different opinion regarding health data sharing after controlling for income and education. The model also includes a dummy variable indicating the presence of chronic diseases. On the one hand, people with chronic diseases may be at a higher risk of data misuse, but on the other hand, they may be more likely to benefit from digitally supported medical progress. In fact, interest in sharing data appears to be 0.2 points higher for people with chronic physical diseases.

The second model adds comfort with digital technologies. As in comparable studies from other countries, there are significant effects. People who find it easy to use digital technology are more open to sharing their health data. However, the effect disappears when the trust variables are included (model 3). Trust turns out to be a very strong influencing factor. Trust in science has the strongest influence. This is particularly noteworthy because the trust indicator does not specifically address the EHR or the institutions behind it but science as a whole. Controlling for socioeconomic parameters, a person who mistrusts the scientific system has a willingness to share that is more than 2 points lower on a scale of 5 than a person who fully trusts the scientific system. In addition, trust in the big technology firms also has a highly significant influence on the hypothesized direction. The strong influence of trust can be seen directly in the sharp increase in the fit of the model (*R*²). The model also includes a proxy for altruistic tendencies in the form of a general willingness to donate (eg, money). The dummy for people who donate on a regular basis is significant. In the last model, motivations and concerns that may be associated with data sharing were included. However, the last model should be interpreted with caution. Because the wording of the questions in the independent variables refers to data sharing, the coefficients may be biased by self-reference (eg, if you care about doing good for society by sharing your data, you will more be willing to share). Nevertheless, the coefficients provide important clues about the motives that drive data sharing. The multivariate results show an interplay between altruistic motives (doing good for society) and cost-benefit considerations (potential benefits for personal and fear of data breaches that expose personal health data to the public).

**Table 2.** Willingness to share data of electronic health record for research purposes—linear regression model^a^.

	Model 1 (n=828)		Model 2 (n=828)		Model 3 (n=828)		Model 4 (n=828)	
	Coefficient	*P* value	Coefficient	*P* value	Coefficient	*P* value	Coefficient	*P* value
Migrant (0/1^b^)	−0.110	.57	−0.116	.54	−0.0190	.92	−0.0431	.83
Gender (reference=woman and 1 nonbinary person)								
Man (0/1)	0.153	.09	0.120	.19	0.0947	.24	0.140	.07
Age (years)	−0.0530^c^	.002	−0.0543^c^	.002	−0.0254	.11	−0.0133	.38
Age² (years)	0.000515^c^	.002	0.000542^c^	.001	0.000293	.06	0.000163	.27
Educational level (reference=medium)								
Low (0/1)	−0.233	.05	−0.217	.07	−0.184	.08	−0.151	.13
High school (0/1)	−0.321^d^	.02	−0.326^d^	.01	−0.319^c^	.01	−0.251^d^	.03
University (0/1)	0.00286	.98	−0.0100	.93	−0.0246	.83	−0.00558	.95
Income^e^	0.000130^c^	.002	0.000128^c^	.002	0.000136^f^	<.001	0.000116^f^	.001
Chronic disease (0/1)	0.201^d^	.03	0.206^d^	.03	0.118	.19	0.0804	.31
Mental illness (0/1)	0.0810	.56	0.0855	.54	0.0441	.73	−0.00517	.96
Rare internet use (0/1)^g^	—^h^	—	−0.229	.09	−0.235	.07	−0.0940	.42
Digital openness^i^	—	—	0.107^d^	.02	0.0330	.42	0.0379	.33
Trust in ...^j^								
Sciences	—	—	—	—	0.417^f^	<.001	0.191^f^	<.001
Big technology companies	—	—	—	—	0.181^f^	<.001	0.0948^d^	.02
Donate regularly (0/1)^k^	—	—	—	—	0.183^d^	.026	0.0474	.54
Motives for data sharing								
Doing good for society^l^	—	—	—	—	—	—	0.206^f^	<.001
Improve my health^m^	—	—	—	—	—	—	0.293^f^	<.001
Afraid of data breach (0/1)^n^	—	—	—	—	—	—	−0.206^c^	.007
Constant	4.307^f^	<.001	3.927^f^	<.001	1.448^c^	.002	0.571	.19

a Own calculations, weighted results. Model 1: *R*2=0.059; model 2: *R*2=0.072; model 3: *R*2=0.270; and model 4: *R*2=0.430.

b 0/1 points to dummy variables.

c *P*<.01.

d *P*<.05.

e Net household equivalence income, imputed (5 times).

f *P*<.001.

g Once a week or less.

h Not applicable; regression model with fewer covariates.

i Using digital hardware and software feels easy for me (1.5=agree totally).

j Trust (1.5=trust totally) in technology companies like Google, Facebook, Apple or in sciences.

k Donate for charity on a regular basis (monthly or yearly).

l Subjective importance that with data sharing I am doing good for society (1.5=very important).

m Subjective importance that with data sharing I profit regarding my personal health.

n I am worried that my data will be made public (0=disagree or 1=agree 1).

## Discussion

### Principal Findings

The results of this study shed light on attitudes toward the use and sharing of EHRs for research purposes in Germany, particularly in the context of the new legislation based on the opt-out mechanism. Overall, the study reveals a nuanced picture of the German population’s attitudes. The empirical results show an openness to EHRs (about half of the nonusers express a desire to use their EHR, signaling significant potential for increased adoption). At the same time, a significant share of the population remains skeptical or undecided. The overall openness is in line with international research regarding interest in EHR [12]. Given these high levels of interest, the introduction of an opt-out model does not seem unjustified. However, especially a large fraction of undecided people need to be properly informed about the consequences of having their health data stored electronically. While there is general support for sharing health data, with 43.4% (n=432) of respondents indicating a willingness to do so, a significant portion (n=225, 22.6%) remain opposed, and a large number are undecided. The results indicate a significantly lower willingness to share health data than has been measured in comparable studies in Germany [15,33]. Possible explanations for these differences are (1) different timing: other studies collected their data at a time when there was no legal framework for health data sharing and therefore risks, etc, had not yet been widely discussed in public. (2) Methodological: this study is based upon a telephone survey rather than web-based and explicitly allows people to have no specific attitude by including categories like refuse to answer, do not know, and neither nor. Last but not least, the comparatively low willingness to share health data in this study compared to international research [27,28] may be due to the emphasis on the personal character of the data potentially shared. The ambivalent results highlight the need for careful consideration of how the opt-out mechanism is implemented and communicated. Although there is openness in principle, “soft paternalism” must be viewed critically precisely because of the high proportion of undecided citizens. In contrast to the principle of informed consent, in which the processes and risks of data sharing have to be explained before citizens allow data sharing, an opt-out solution presupposes that the population is sufficiently informed in other ways. This becomes even more clear after considering attitudes toward the opt-out mechanism, which 44.7% (n=438) disapprove. Especially people who are positive about EHRs and the sharing of their health data reject the opt-out mechanism proposed by the legislator more often. Presumably, “choice architecture” is seen as too much interference in their own decisions.

The study also confirms a digital divide, as socioeconomic factors significantly influence both the willingness to use EHRs and the willingness to share personal health data. The results are in line with international research findings [16,30]. Higher income, education, and digital literacy are associated with greater openness, while those with lower socioeconomic status are more reluctant. However, socioeconomic factors are not the most important in explaining openness to data sharing. Trust plays a critical role in determining the willingness to share health information. While other studies have pointed to the role of trust as a significant factor [15,40,44,45], this study shows that institutional trust has a decisive influence compared to other factors. The influence of socioeconomic variables, as well as other factors such as digital literacy, diminishes when trust variables are taken into account, highlighting trust as a fundamental element in the acceptance of digital health initiatives. The observed socioeconomic disparities reinforce the need for targeted interventions and information to ensure equitable access and participation in digital health.

### Policy Implications

The paradigm shift to an opt-out system is expected to increase participation rates because it relies on passive inclusion rather than active consent. However, this comes at the cost of potentially creating a population of uninformed and passive citizens who are enrolled in the system without fully understanding the implications. This raises critical concerns about consumer autonomy and challenges the notion of the autonomous consumer who actively engages with—and consents to—data-sharing practices. With a large portion of the population still undecided about EHR adoption and data sharing, the need for population-wide education becomes even more apparent. Ideally, all citizens, regardless of their socioeconomic background, should be able to make informed and autonomous decisions about their participation. The study also shows the complex mechanisms of the digital divide, with those in higher socioeconomic positions being more open to the adoption of EHRs but also more likely to oppose the opt-out system. This suggests that the paradigm shift is perceived as a restriction of autonomy. Those with greater resources and digital literacy want to make informed choices and therefore resist mechanisms that limit their freedom of choice. While those with greater resources are likely to be able to opt out if they perceive the risks of data sharing to be too high, they could trigger serious political resistance to the opt-out mechanism. In contrast, individuals with lower socioeconomic status may be less able to navigate these systems and therefore are more vulnerable to passive inclusion.

The transition to an opt-out system for EHRs in Germany marks a significant policy shift with the potential to transform the health sector. However, for this shift to be successful, it is essential to address the underlying concerns of the population, particularly around data security, autonomy, and the digital divide. Continuous monitoring of the implementation of the opt-out system will be crucial to keep and build trust within the population.

### Limitations and Further Research Questions

In contrast to web-based surveys, which are often used in EHR research, the CATI method used here avoids many problems of self-reporting, sample bias, and inference [48,49]. However, the quality of data collected through telephone interviews also experiences low response rates. It seems highly plausible that nonresponse is not random. People with lower levels of trust and willingness are supposedly less likely to participate in the survey. Thus, despite the weighting procedure applied, the sample may be biased toward more trusting people. This problem is common to other data used in comparable research.

Another limitation concerns the correct understanding of the EHR in Germany. During the field phase, little more than 1% of the insured population opted for the EHR. It is possible that many respondents do not have a proper understanding of the EHR or have no knowledge at all. The survey provides some evidence in this direction. A question about knowledge of the EHR reveals that 13.9% (n=139) have never heard of it. For this group, the evaluation of data sharing is probably less substantiated. However, at the same time, a large fraction says that they have heard of the EHR (n=720, 71.7%) or even use it (n=89, 8.9%). On the one hand, these results support the assertion that a large proportion of the sample has a basic understanding of the EHR. During the field phase, there was also an extensive media discourse about the EHR, as the Gesundheitsdatennutzungsgesetz was being discussed in parliament in December 2023. On the other hand, the self-reported use of the EHR does not match the official statistics. Some respondents probably confused the EHR with other apps provided by their health insurance. This problem of overreporting has also been observed in other studies [15].

The study focuses on the interest in using the EHR and the willingness to share health data. At the time of the field phase, EHR adoption was very low, and a legal framework for health information exchange was in its formative stages. Research has already shown that there is a gap between attitudes and actual behavior [12,42]. So the results have to be interpreted with some caution. In addition, the high proportion of undecided respondents indicates that reported attitudes are not very salient and therefore susceptible to survey effects. However, other studies share this disadvantage. Moreover, the consequences of limited interest in EHR do not only affect surveys but also have far-reaching practical implications, especially when using opt-out regulations.

Future research should focus on the gap between interest in using the EHR and actual behavior. With a focus on Germany, the potential burdens of adoption should be analyzed in more detail. With regard to trust, the potential ways to build trust within the health care system without relying too much on general institutional trust also point to new research directions. Furthermore, there are interesting research questions regarding self-responsibility in a system that becomes largely based on passive consent (opt-out).

## Abbreviations

CATI: computer-assisted telephone interview
EHR: electronic health record

## References

[1] Elektronische Patientenakte bringt Entbürokratisierung und entlastet Ärzte und Pflegekräfte [Web page in German]. Bundesministerium für Gesundheit.

[2] (2021). Digitalisierung für Gesundheit: Ziele und Rahmenbedingungen eines dynamisch lernenden Gesundheitssystems [Web page in German]. Gutachten.

[3] Strotbaum V, Pobiruchin M, Schreiweis B, Wiesner M, Strahwald B (2019). Your data is gold—data donation for better healthcare?. it - Information Technology.

[4] Lupton D (2018). Digital Health.

[5] Caspar J (2023). Wir Datensklaven [Book in German].

[6] Matzat U, van Ingen E, Maasen S, Passoth JH, Maasen S, Passoth JH (2020). Soziologie des Digitalen—Digitale Soziologie.

[7] Merkel S, Hess M (2020). The use of internet-based health and care services by elderly people in Europe and the importance of the country context: multilevel study. JMIR Aging.

[8] Thaler RH, Sunstein C (2009). Nudge Improving Decisions about Health, Wealth and Happiness.

[9] Sunstein CR (2015). Choosing Not to Choose: Understanding the Value of Choice.

[10] Hoerbst A, Kohl CD, Knaup P, Ammenwerth E (2010). Attitudes and behaviors related to the introduction of electronic health records among Austrian and German citizens. Int J Med Inform.

[11] Kruse CS, Stein A, Thomas H, Kaur H (2018). The use of electronic health records to support population health: a systematic review of the literature. J Med Syst.

[12] Crameri KA, Maher L, Van Dam P, Prior S (2022). Personal electronic healthcare records: what influences consumers to engage with their clinical data online? A literature review. Health Inf Manag.

[13] Zheng H, Jiang S (2022). Frequent and diverse use of electronic health records in the United States: a trend analysis of national surveys. Digit Health.

[14] Seifert A (2024). Nur wenige Deutsche nutzen bislang die Elektronische Patientenakte [Web page in German]. mdr.

[15] Haug S, Schnell R, Raptis G, Dotter C, Weber K (2024). Wissen und Einstellung zur Speicherung und Nutzung von Gesundheitsdaten: Ergebnisse einer Bevölkerungsbefragung [Article in German]. Z Evid Fortbild Qual Gesundhwes.

[16] Kim EH, Kim Y Digital divide: use of electronic personal health record by different population groups.

[17] Halmdienst N, Pruckner GJ, Winter-Ebmer R (2023). Complexities of health and acceptance of electronic health records for the Austrian elderly population. Eur J Health Econ.

[18] Paccoud I, Baumann M, Le Bihan E (2021). Socioeconomic and behavioural factors associated with access to and use of personal health records. BMC Med Inform Decis Mak.

[19] Henkenjohann R (2021). Role of individual motivations and privacy concerns in the adoption of German electronic patient record apps—a mixed-methods study. Int J Environ Res Public Health.

[20] Griesser A, Bidmon S (2022). A process related view on the usage of electronic health records from the patients’ perspective: a systematic review. J Med Syst.

[21] Stablein T, Hall JL, Pervis C, Anthony DL (2015). Negotiating stigma in health care: disclosure and the role of electronic health records. Health Sociol Rev.

[22] Kalckreuth N, Prümper AM, Feufel MA (2023). The influence of health data on the use of the electronic health record (EHR): a mixed methods approach.

[23] Eriksson-Backa K, Hirvonen N, Enwald H, Huvila I (2021). Enablers for and barriers to using My Kanta— a focus group study of older adults’ perceptions of the National Electronic Health Record in Finland. Inform Health Soc Care.

[24] Greenberg AJ, Falisi AL, Finney Rutten LJ (2017). Access to electronic personal health records among patients with multiple chronic conditions: a secondary data analysis. J Med Internet Res.

[25] Nolte D (2021). Vom Daten-Grab zum Daten-Schatz? Strukturierte Behandlungsprogramme (Disease Management Programme) für chronisch Kranke. Zugluft.

[26] Caine K, Kohn S, Lawrence C, Hanania R, Meslin EM, Tierney WM (2015). Designing a patient-centered user interface for access decisions about EHR data: implications from patient interviews. J Gen Intern Med.

[27] Skatova A, Goulding J (2019). Psychology of personal data donation. PLOS ONE.

[28] Howe N, Giles E, Newbury-Birch D, McColl E (2018). Systematic review of participants’ attitudes towards data sharing: a thematic synthesis. J Health Serv Res Policy.

[29] Kalkman S, van Delden J, Banerjee A, Tyl B, Mostert M, van Thiel G (2022). Patients’ and public views and attitudes towards the sharing of health data for research: a narrative review of the empirical evidence. J Med Ethics.

[30] Hirst Y, Stoffel ST, Brewer HR, Timotijevic L, Raats MM, Flanagan JM (2023). Understanding public attitudes and willingness to share commercial data for health research: survey study in the United Kingdom. JMIR Public Health Surveill.

[31] Kühnel E, Wilke F, Heckes KT, Lorke M, Siegler M, Wrona KJ (2025). Soziotechnische Transformationen im Sozial- und Gesundheitswesen: kollaborativ, divers, barrierefrei und sozialräumlich.

[32] Richter G, Borzikowsky C, Hoyer BF, Laudes M, Krawczak M (2021). Secondary research use of personal medical data: patient attitudes towards data donation. BMC Med Ethics.

[33] Richter G, Trigui N, Caliebe A, Krawczak M (2024). Attitude towards consent-free research use of personal medical data in the general German population. Heliyon.

[34] Lee H, Kim S, Couper MP, Woo Y (2019). Experimental comparison of PC web, smartphone web, and telephone surveys in the new technology era. Soc Sci Comput Rev.

[35] Milton AC, Ellis LA, Davenport TA, Burns JM, Hickie IB (2017). Comparison of self-reported telephone interviewing and web-based survey responses: findings from the Second Australian Young and Well National Survey. JMIR Ment Health.

[36] Moon LA (2017). Factors influencing health data sharing preferences of consumers: a critical review. Health Policy Technol.

[37] Karampela M, Ouhbi S, Isomursu M (2019). Connected health user willingness to share personal health data: questionnaire study. J Med Internet Res.

[38] Weng C, Friedman C, Rommel CA, Hurdle JF (2019). A two-site survey of medical center personnel’s willingness to share clinical data for research: implications for reproducible health NLP research. BMC Med Inform Decis Mak.

[39] Broekstra R, Aris-Meijer JL, Maeckelberghe ELM, Stolk RP, Otten S (2022). Motives for withdrawal of participation in biobanking and participants’ willingness to allow linkages of their data. Eur J Hum Genet.

[40] Mählmann L, Schee gen. Halfmann S, von Wyl A, Brand A (2018). Attitudes towards personal genomics and sharing of genetic data among older Swiss adults: a qualitative study. Public Health Genomics.

[41] Braunack-Mayer A, Fabrianesi B, Street J (2021). Sharing government health data with the private sector: community attitudes survey. J Med Internet Res.

[42] Benevento M, Mandarelli G, Carravetta F (2023). Measuring the willingness to share personal health information: a systematic review. Front Public Health.

[43] Kühnel E, Wilke F (2025). Health data sharing in Germany: individual preconditions, trust and motives. Front Public Health.

[44] Weitzman ER, Kaci L, Mandl KD (2010). Sharing medical data for health research: the early personal health record experience. J Med Internet Res.

[45] Maiorana A, Steward WT, Koester KA (2012). Trust, confidentiality, and the acceptability of sharing HIV-related patient data: lessons learned from a mixed methods study about Health Information Exchanges. Implement Sci.

[46] Meyer S, Ward P, Coveney J, Rogers W (2008). Trust in the health system: an analysis and extension of the social theories of Giddens and Luhmann. Health Sociol Rev.

[47] Wilke F, Kühnel E (2024). Eine Befragung zur Bereitschaft zum Teilen von Gesundheitsdaten in Deutschland—Einflussfaktoren, Präferenzen und Motive.

[48] McPhee C, Barlas F, Brigham N, Darling J, Dutwin D (2022). Data quality metrics for online samples: considerations for study design and analysis. https://aapor.org/wp-content/uploads/2023/02/Task-Force-Report-FINAL.pdf.

[49] Pokorny S, Hirndorf D (2024). Online, offline oder beides? Umfragemethoden im Praxistest [Article in German]. Analysen Argumente.

